# Myocardial infarction type 1 is frequent in refractory out-of-hospital cardiac arrest (OHCA) treated with extracorporeal cardiopulmonary resuscitation (ECPR)

**DOI:** 10.1038/s41598-020-65498-9

**Published:** 2020-05-21

**Authors:** D. Duerschmied, V. Zotzmann, M. Rieder, X. Bemtgen, P. M. Biever, K. Kaier, G. Trummer, C. Benk, H. J. Busch, C. Bode, T. Wengenmayer, P. Stachon, C. von zur Mühlen, D. L. Staudacher

**Affiliations:** 1 Department of Medicine III (Interdisciplinary Medical Intensive Care), Medical Center, Faculty of Medicine, University of Freiburg, Freiburg, Germany; 2 Department of Cardiology and Angiology I, Heart Center, University of Freiburg, Freiburg, Germany; 3 Institute for Medical Biometry and Statistics, Faculty of Medicine, University of Freiburg, Freiburg, Germany; 4 Department of Cardiovascular Surgery, Heart Center Freiburg University, University of Freiburg, Freiburg, Germany; 5 Department of Emergency Medicine, University Hospital of Freiburg, Faculty of Medicine, University of Freiburg, Freiburg, Germany

**Keywords:** Interventional cardiology, Cardiac device therapy

## Abstract

Extracorporeal cardiopulmonary resuscitation (ECPR) is a last resort treatment option for refractory cardiac arrest performed in specialized centers. Following consensus recommendations, ECPR is mostly offered to younger patients with witnessed collapse but without return of spontaneous circulation (ROSC). We report findings from a large single-center registry with 252 all-comers who received ECPR from 2011–2019. It took a median of 52 min to establish stable circulation by ECPR. Eighty-five percent of 112 patients with out-of-hospital cardiac arrest (OHCA) underwent coronary angiography, revealing myocardial infarction (MI) type 1 with atherothrombotic vessel obstruction in 70 patients (63% of all OHCA patients, 74% of OHCA patients undergoing coronary angiography). Sixty-six percent of 140 patients with intra-hospital cardiac arrest (IHCA) underwent coronary angiography, which showed MI type 1 in 77 patients (55% of all IHCA patients, 83% of IHCA patients undergoing coronary angiography). These results suggest that MI type 1 is a frequent finding and - most likely - cause of cardiac arrest (CA) in patients without ROSC, especially in OHCA. Hospital survival rates were 30% and 29% in patients with OHCA and IHCA, respectively. According to these findings, rapid coronary angiography may be advisable in patients with OHCA receiving ECPR without obvious non-cardiac cause of arrest, irrespective of electrocardiogram analysis. Almost every third patient treated with ECPR survived to hospital discharge, supporting previous data suggesting that ECPR may be beneficial in CA without ROSC. In conclusion, interventional cardiology is of paramount importance for ECPR programs.

## Introduction

Current treatment algorithms for cardiac arrest (CA) advocate a prompt treatment of the underlying cause^1^. In patients with ST-segment elevation myocardial infarction (STEMI), revealed by 12-lead electrocardiogram analysis after return of spontaneous circulation (ROSC), an immediate coronary angiography with percutaneous coronary intervention (PCI) improves outcome^2^. Registry data on out-of-hospital cardiac arrest (OHCA) suggest a coronary cause of CA in 29% to 43% of patients presenting without ST-segment elevation^3,4^. OHCA patients without STEMI might therefore also benefit from an early coronary angiography.

**Figure 1:**
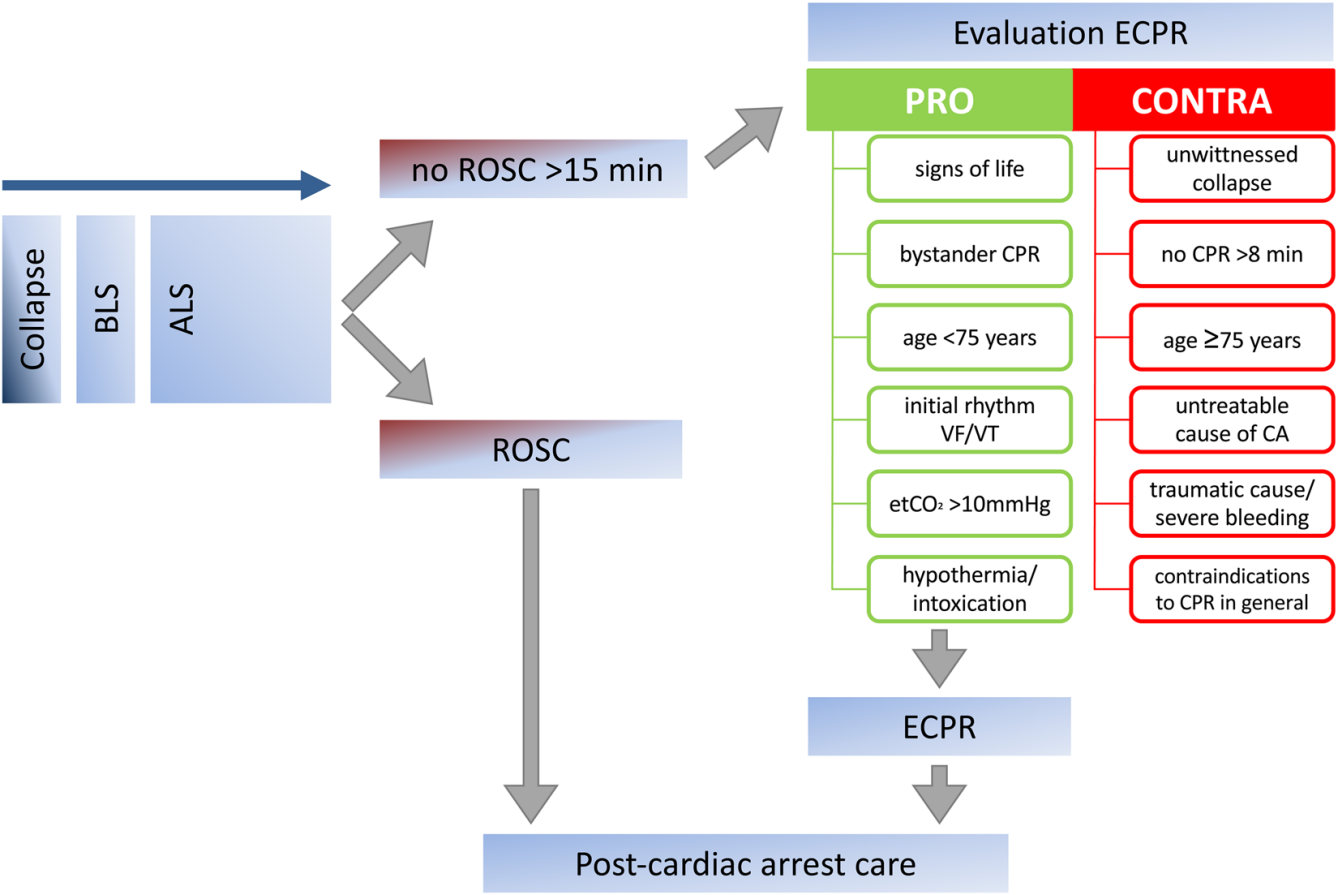
Algorithm for decision making pro or contra extracorporeal cardiopulmonary resuscitation (ECPR) initiation in refractory cardiac arrest. BLS – basic life support, ALS – advanced life support, ROSC – return of spontaneous circulation, etCO_2_ – end-tidal CO_2_, VF – ventricular fibrillation, VT – ventricular tachycardia, CA – cardiac arrest.

However, more information about patients receiving extracorporeal cardiopulmonary resuscitation (ECPR) is needed, because this is a selected patient cohort^5^. In refractory CA, ECPR is considered an ultima ratio treatment option if ROSC cannot be achieved within 30 min of conventional CPR. According to consensus recommendations, ECPR should be considered in case of refractory CA if favorable prognostic factors are present and unfavorable factors are absent^6–8^. Figure 1 shows the decision algorithm used in our center to determine a possible indication or contra-indication for initiating ECPR. Notably, patients of advanced age (≥75 years old) will not routinely receive ECPR. A no-flow time of >8 min, an unwitnessed collapse, and an evident untreatable cause of CA are other factors that would not prompt ECPR initiation, but rather lead to termination of resuscitation efforts. Favorable factors are signs of life under CPR, an initially shockable rhythm, and sufficient end-tidal CO_2_. Decision-making pro or contra initiation of ECPR is recommended after 15 min of advanced life support in our and many other centers to minimize low-flow time^9^.

Together, these criteria are meant to ensure that a resource-intensive and highly invasive procedure such as ECPR is reserved to CA patients without ROSC, but still rather favorable prognosis. ECPR patients are therefore a very distinct patient cohort, defined by criteria that also favor the selection of patients with a treatable, most often cardiac cause of CA. Therefore, we set out to further investigate logistics and outcome of ECPR patients using an all-comers single-center registry.

## Methods

### Study setting

Presented data derive from a single-center retrospective registry analysis with ethics approval (Ethics Committee of Albert-Ludwigs University of Freiburg, file number 151/14 and 533/19). Only anonymized clinical data were obtained retrospectively from a cardiac arrest cohort with many non-survivors and the ethics committee waived the need for individual written informed consent. All methods were performed in accordance with relevant guidelines and regulations. This study includes all patients receiving ECPR at our tertiary referral university hospital between May 2011 and May 2019. ECPR was defined as VA-ECMO implantation during continuous CPR without ROSC or as VA-ECMO implantation within the first 20 min after ROSC with uncontrollable hemodynamic instability^10^. All VA-ECMO cases treated at our institution were detected by a computerized search for the German OPS-codes (*Operationen- und Prozedurenschlüssel*) for VA-ECMO (8–852.30–8–852.30e), followed by manual review on case-by-case basis. All patients treated at the interdisciplinary medical intensive care unit were considered for this retrospective registry analysis, excluding patients cannulated for VA-ECMO in the operation theatre and treated at a surgical intensive care unit.

### Local ECPR algorithm

Since 2011, all patients with IHCA without ROSC after 15 minutes are routinely screened for an indication for ECPR. As defined by our standard operating procedures, unwitnessed cardiac arrest, prolonged duration of CPR without signs of life (breathing, swallowing etc.), a non-shockable initial rhythm, life-threatening bleeding and advanced age ≥75 years are considered relative contraindications for ECMO cannulation (Fig. 1). Final decision to cannulate is driven by a team decision at the bedside including at least two physicians, a perfusionist, and two nurses. Local standard operating procedure suggests cannulation for ECPR after IHCA on-site or in the cardiac catheterization laboratory, whichever results in shorter low-flow durations. For OHCA, emergency medical services personnel are encouraged to transport patients without ROSC with ongoing mechanical chest compressions to our center, where the patients are screened for ECPR. The same contraindications apply as for IHCA patients. OHCA patients were either routed to the emergency room or the cardiac catheterization laboratory, according to the presumed cause for collapse, non-cardiac or cardiac, respectively^11^. Screening for ECPR and cannulation were then performed in the emergency room or the catheterization laboratory, whichever destination had been chosen. Starting in August 2018, an out-of-hospital ECPR service was implemented, offering on-site out-of-hospital ECPR during working hours (10 patients in this cohort).

### ECMO cannulation and maintenance

Local standard operating procedures suggest cannulation for ECPR to be performed in Seldinger’s technique by two experienced intensivists and one perfusionist. SCPC (Sorin Centrifugal Pump Console, LivaNova, London, United Kingdom) or Cardiohelp (Maquet Getinge Group, Rastatt, Germany) systems could be used. Typical venous (draining) cannulas were 21–23 Fr (French = Charrière) in diameter and 55 cm of length while arterial (returning) cannulas were 15–17 Fr, 23 cm (both HLS cannula, Maquet Getinge Group, Rastatt, Germany). For patients without life-threatening bleeding, anticoagulation was provided by intravenous administration of unfractionated heparin aiming at a partial thromboplastin time of 50–60 sec. The management of vasopressors and fluid therapy was driven by clinical judgement of the ECMO-experienced intensivist in charge and has been reported earlier^12,13^. Notably, more liberal fluid resuscitation with albumin (in a 1:2 ratio with balanced crystalloid solution) was introduced in 2016.

### Coronary angiography after ECPR

Early detection and treatment of the cause for collapse was attempted in all ECPR cases. A coronary angiography was therefore performed in all patients with a presumed cardiac cause. In case of presumed non-cardiac cause and non-conclusive findings in ultrasound and computed tomography studies, a coronary angiography is advertised by local standard operating procedures. Intervention of a coronary stenosis detected by angiography was performed if recommended by current guidelines^14^. Types of MI were primarily diagnosed by the responsible interventionalist, but then adjudicated by an experience, independent cardiologist off-line^15^. MI Type 1 was adjudicated in patients with myocardial necrosis (defined by troponin T above the 99^th^ percentile upper reference limit) if a total or sub-total atherothrombotic vessel obstruction was present and treated by PCI. MI Type 2 was adjudicated in patients with myocardial necrosis if a hemodynamically relevant coronary artery stenosis ≥75% without total or sub-total vessel obstruction was present and treated by PCI. All other cases of CA were allocated to a non-MI cause of arrest.

### Data analysis

For data analysis, Prism (version 7, GraphPad) was used and a p-value of <0.05 was considered statistically significant. All data are given as [mean ± standard deviation] if not stated otherwise. Unpaired t test (if Gaussian distribution was assumed as tested by Kolmogorov-Smirnov normality test) or Mann-Whitney test (in cases where normal distribution could not be assumed) were used to compare values from OHCA versus IHCA patients. Normal distribution was assumed for the parameters SAPS II, pH, lactate, hemoglobin, K^+^, contrast agent volume, and number of vessels treated. Fisher’s exact test was used to interpret contingency tables.

## Results

### Patient characteristics

**Figure 2:**
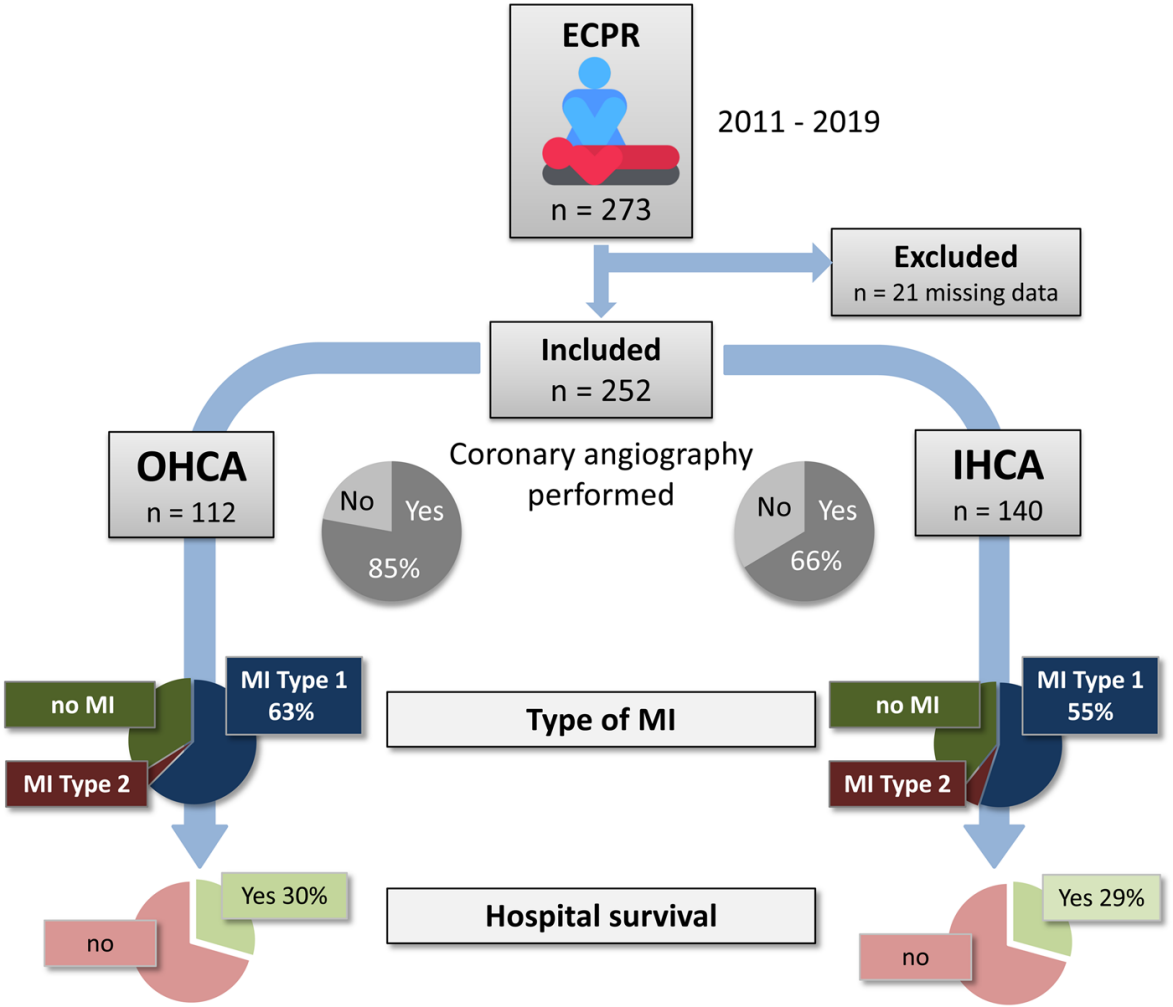
Recruitment of 273 extracorporeal cardiopulmonary resuscitation (ECPR) all-comers in our single-center registry allowed the analysis of 112 patients with out-of-hospital cardiac arrest (OHCA) and 140 patients with in-hospital cardiac arrest (IHCA) after exclusion of 21 incomplete data sets. Rates of performed coronary angiography, types of myocardial infarction (MI), and hospital survival are shown.

Out of 273 all-comer cases receiving ECPR during the observational period, 252 complete data sets could be retrieved from the registry (Fig. 2). Patients receiving ECPR for OHCA were significantly younger than patients receiving ECPR for IHCA, with an overall mean age of 59 years (Table 1). Other baseline characteristics were equally distributed between the OHCA and IHCA groups, but underlying lung disease, hypertension, diabetes mellitus, chronic kidney disease, and dyslipidemia were more frequent in IHCA patients.

**Table 1:** Baseline characteristics.

	**All (n** = **252)**	**OHCA (n** = **112)**	**IHCA (n** = **140)**	***p*** **OHCA vs. IHCA**
Age (y)	59.0 ± 14.3	54.8 ± 14.3	62.5 ± 13.5	**<0.0001**
Male	186 (73.8%)	88 (78.6%)	98 (70.0%)	0.1496
BMI (kg/m²)	27.0 ± 5.1	26.7 ± 4.7	27.2 ± 5.3	0.5837
Lung disease^a^	35 (13.9%)	9 (8%)	26 (18.6%)	**0.0175**
Hypertension	98 (38.9%)	31 (27.7%)	67 (47.9%)	**0.0012**
Diabetes mellitus	58 (23.0%)	12 (10.7%)	46 (32.9%)	**<0.0001**
CAD	148 (58.7%)	59 (52.7%)	89 (63.6%)	0.0945
PAD	17 (6.7%)	6 (5.4%)	11 (7.9%)	0.4624
Chronic kidney disease	42 (16.7%)	10 (8.9%)	32 (22.9%)	**0.0036**
Dyslipidemia	56 (22.2%)	18 (16.1%)	38 (27.1%)	**0.0470**
Smoking	68 (27.0%)	24 (21.4%)	44 (31.4%)	0.0872
Shockable rhythm	120 (47.6%)	66 (58.9%)	54 (38.6%)	**0.0015**
No-flow time (minutes/ median (min-max); mean ± SD)	0 (0–30); 1.8 ± 4.2	0 (0–30); 3.5 ± 5.6	0 (0–15); 0.6 ± 2.2	**<0.0001**
Time to ECMO (minutes/ median (min-max); mean ± SD)	52 (10–150) 56.2 ± 28.0	64 (15–150); 67.4 ± 29.0	45 (10–140); 47.3 ± 23.8	**<0.0001**
Initial pH	7.15 ± 0.2	7.07 ± 0.2	7.21 ± 0.2	**<0.0001**
Initial pCO_2_ (mmHg)	49.4 ± 23.4	52.6 ± 23.0	47.2 ± 23.6	0.1144
Initial pO_2_ (mmHg)	109.6 ± 95.5	91.1 ± 85.3	123.8 ± 101.2	0.1039
Initial lactate (mmol/L)	11.0 ± 8.6	11.8 ± 4.9	10.5 ± 10.5	0.2972
Initial hemoglobin (g/dL)	12.0 ± 3.3	13.2 ± 2.6	10.9 ± 3.5	**0.0035**
Initial K^+^ (mmol/L)	4.7 ± 1.2	4.8 ± 1.3	4.6 ± 1.0	0.3730
Initial SAPS II	79.4 ± 12.5	78.8 ± 11.4	79.8 ± 13.3	0.5221

BMI – Body mass index; ^a^Lung disease: history of COPD, asthma, lung fibrosis, cystic fibrosis; CAD - history of coronary artery disease; PAD - history of peripheral arterial disease; SAPS - Simplified acute physiology score, bold *p* values denote statistically significant differences.

Patients presented with a shockable rhythm in 59% and 38% in OHCA and IHCA, respectively. While the median no-flow time was <1 min, we observed a wide range from 0 up to 30 min (in one OHCA patient who did not survive to hospital discharge). No-flow time was significantly longer in OHCA than in IHCA. The median time from collapse to start of ECMO, i.e. established extracorporeal circulation, was 64 min in OHCA and 45 min in IHCA patients.

Initial – first measured – pH was 7.1 in OHCA and 7.2 in IHCA. As expected from a patient cohort with refractory CA, initial lactate levels were relatively high with a mean of 11.8 mmol/L in OHCA and 10.5 mmol/L in IHCA. Also, reflecting the severity of disease, initial SAPS II was high in both groups.

### Patient survival

Mean duration of VA-ECMO was 131 h, without significant differences between OHCA and IHCA patients. Mean ECMO flow rate in the entire cohort was 3.6 l/min after 24 h. Twenty-nine percent of all patients survived to hospital discharge – without significant differences between the groups.

### Coronary angiography findings

In total, a coronary angiography was performed in 75% of cases, with a higher frequency in OHCA (85%, Fig. 2) than in IHCA (66%). Most likely, this can be explained by other causes of CA that were suspected, diagnosed, and promptly treated in patients with IHCA as compared to OHCA. For instance, relevant lung disease was more than twice as frequent in IHCA than in OHCA patients. IHCA patients were also older and presented with other comorbidities, including chronic kidney disease (Table 1) and initially increased markers of renal dysfunction (first values of blood urea nitrogen and creatinine measured after ECPR were significantly higher in IHCA patients, Table 2).

**Table 2:** Outcome data.

	**All (n** = **252)**	**OHCA (n** = **112)**	**IHCA (n** = **140)**	***p*** **OHCA vs. IHCA**
VA-ECMO duration (h)	130.7 ± 587.8	151.6 ± 835.4	114.1 ± 263.0	0.6176
Hospital Survival	74 (29.4%)	33 (29.5%)	41 (29.3%)	>0.9999
First blood urea nitrogen after ECPR (mg/dL)	52.8 ± 34.6	42.1 ± 24.8	62.2 ± 39.1	**<0.0001**
First creatinine after ECPR (mg/dL)	1.6 ± 1.2	1.4 ± 0.8	1.8 ± 1.4	**0.0107**
Acute kidney injury	160 (63.5%)	69 (61.6%)	91 (65.0%)	0.6004
Hemodialysis	58 (23.0%)	22 (19.6%)	36 (25.7%)	0.2931

Bold *p* values denote statistically significant differences.

MI Type 1 was diagnosed in 63% of OHCA patients and 55% of IHCA patients, a difference that was not statistically significant (Table 3, Fig. 2). Non-occluded, but severely stenosed coronary arteries were diagnosed in less than 5% of all cases (classified as MI Type 2), without a significant difference between the groups. As expected in a CA patient cohort, door-to-balloon time was relatively long (46 min). Surprisingly, IHCA patients had a significantly longer door-to-balloon time (53 min) than OHCA patients (39 min). The most likely reason for this difference was the significantly higher rate of shockable rhythm in OHCA patients, indicating a cardiac cause of arrest and prompting more rapid coronary angiography and PCI. In IHCA, other causes of CA were often suspected and differential diagnostic procedures performed first.

**Table 3:** Coronary angiography results.

	**All (n** = **252)**	**OHCA (n** = **112)**	**IHCA (n** = **140)**	***p*** **OHCA vs. IHCA**
Coronary angiography performed within 24 h	188 (74.6%)	95 (84.8%)	93 (66.4%)	**0.0008**
ECMO to coronary angiography time (h)	0.6 ± 1.74	0.5 ± 0.8	0.7 ± 2.4	0.4112
Contrast agent volume (mL)	295.2 ± 178.5	236.6 ± 125.6	353.8 ± 203.4	**<0.0001**
MI Type 1 treated with PCI	147 (58.3%)	70 (62.5%)	77 (55.0%)	0.2489
MI Type 2 treated with PCI	12 (4.8%)	4 (3.6%)	8 (5.7%)	0.5565
Cause of cardiac arrest other than MI	93 (36.9%)	38 (34.0%)	55 (39.3%)	0.4312
Vessels treated (number)	1.5 ± 0.7	1.4 ± 0.6	1.6 ± 0.7	0.925
Left main PCI	48 (23.8%)	18 (16.1%)	30 (29.4%)	**0.0221**
Stents implanted (number)	2.2 ± 2.1	1.9 ± 1.8	2.5 ± 2.3	0.780
Door-to-balloon time (min)	46.0 ± 26.6	39.1 ± 18.5	52.6 ± 31.4	**0.0024**

MI – myocardial infarction; PCI – percutaneous coronary intervention; MI Type 1 – atherothrombotic vessel occlusion; MI Type 2 – MI not caused by atherothrombotic vessel occlusion, but a coronary artery stenosis ≥75%, bold *p* values denote statistically significant differences.

The survival rate of patients with MI Type 1 was similar to the entire cohort. Survival rates of the groups of patients with MI Type 2 (17%) or other cause of CA (26%) were smaller than the survival rate of patients with MI Type 1 (29%), but these differences were not statistically significant (p = 0.513 and p = 0.851, respectively). When interpreting these numbers, the smaller sizes of the groups of patients with MI Type 2 (n = 12) of other cause of CA (n = 83) as compared to the group of patients with MI Type 1 (n = 147) should be considered.

### Kidney injury

We observed acute kidney injury in approximately 64% of all patients and saw no significant difference between OHCA and IHCA patients. Hemodialysis was performed in 23% of all patients (no significant difference between the groups).

The equal frequencies of kidney injury and hemodialysis did not reflect the significantly larger volume of contrast agent used in IHCA patients. Following IHCA, not only was more contrast agent used, but also more vessels were treated and more stents were implanted (both not significant). Notably, significantly more left main stem PCIs were performed in IHCA patients (29%) as compared to OHCA (16%). These results are in line with the observation that IHCA occurred peri-interventionally during especially complex and/ or complicated PCIs in 24%. Out of peri-interventional IHCA cases, 47% required left main PCIs. Therefore, PCI was more complicated in our IHCA cohort, because complex PCI was the cause of IHCA in many cases, but this did not translate into a higher rate of acute kidney injury.

## Discussion

### Immediate coronary angiography after ECPR

Whether early coronary angiography should be performed in all ECPR patients, or only after selection according to interpretation of electrocardiogram and other information as recommended in patients without CA or after ROSC, has not been investigated systematically. For patients with refractory CA without ROSC, current guidelines recommend an urgent coronary angiography only if it appears logistically feasible and if an acute coronary syndrome is suspected^1^. Shockable primary rhythm is associated with both cardiac cause for collapse^16^ and improved outcome in ECPR^17^ and is consequently one of the factors in favor of ECPR initiation^6^. In addition, logistics in a catheterization laboratory appear ideal for rapid, sterile, effective, and safe cannulation to establish VA-ECMO under CPR. It is therefore conceivable that in-hospital ECPR in a cardiac catheterization laboratory followed by immediate coronary angiography may be beneficial^18^.

An analysis of the Paris registry recently showed that 73% of OHCA patients receiving pre-hospital ECPR in the mobile intensive care unit had relevant coronary artery disease^19^. This is in line with earlier data from the French PROCAT II-registry showing that among patients with ROSC after OHCA and without ST-segment elevation, 29% were treated with PCI^3^. PCI was associated with better outcome in these patients. Much higher rates of severe coronary artery disease were found in patients with refractory ventricular fibrillation (up to 84%) in a US registry^20^. Our analysis is the first to examine the rates of MI Types 1 and 2 in ECPR patients after OHCA and IHCA.

The results from the all-comers cohort of ECPR patients treated at our center support the earlier data: Sixty-three percent of all OHCA patients had an MI Type 1 and another 4% had an MI Type 2. This means that two thirds of OHCA patients had severe and ongoing myocardial necrosis, which should be stopped as soon as possible by revascularization to prevent further myocardial scarring according to all current guidelines^14^.

We conclude that immediate coronary angiography should be offered to all ECPR patients without obvious non-cardiac cause of CA. Ideally, CA patients without ROSC and scheduled for ECPR should therefore be transferred into a cardiac catheterization laboratory, where VA-ECMO can be implanted first and coronary angiography can be performed immediately after that. Further diagnostic workup, including whole-body computed tomography, can then be integrated into the post-CA care algorithm (Fig. 1), ideally very early, i.e. directly after coronary angiography^21^. In our center, echocardiography is routinely performed much earlier: during advanced life support in the pre-hospital setting or together with endotracheal tube position control and blood gas analysis as first steps in the cardiac catheterization laboratory as part of a focused abdominal sonography for trauma and focused echocardiography in emergency life support algorithm^6,11–13,22^.

### Survival

Although the initial prognostication for survival by scores such as SAPS II will underestimate the severity of disease in CA patients, because parameters such as blood urea nitrogen or bilirubin have not yet increased shortly after CPR^23^, a mean SAPS II of 79 (Table 1) predicts a disastrous hospital survival rate of <10%^24,25^. In contrast to this poor prognosis, we observed a much higher survival rate in our ECPR patients of 29%, suggesting that ECPR improved survival. Our registry data are however not suitable to provide high-level evidence supporting this statement and a randomized controlled trial is desirable to determine whether ECPR saves lives.

A recent controversy about this question increased the need for such high level of evidence-providing trials, one of which is currently recruiting patients (ClinicalTrials.gov Identifier: NCT03637205). Rather disappointing results from a large French registry showed similar hospital survival rates of 8% in CA both with and without ECPR^26^. It should be noted, however, that the duration of CPR was longer than 30 min in 99% of patients receiving ECPR compared to 77% of patients receiving conventional CPR in this report. This supports the assumption that ECPR was predominantly provided to patients not achieving ROSC – a patient cohort with an expected survival close to 0%. Further illustrating this imbalance between study groups was a more than doubled lactate level (14.5 mmol/L) in ECPR patients compared to patients treated with conventional CPR (6.9 mmol/L). The French study hence contained a relevant bias and should not be interpreted as showing a null effect of ECPR, but rather inform about factors that need to be improved to increase survival, such as patient selection.

Evidence in favor of ECPR was reported from the Brussels group with an improved favorable neurological outcome at 3 months (defined as cerebral performance category 1–2) of 21% in CA patients receiving ECPR as compared to 11% in patients receiving conventional CPR^27^. Hospital survival was 23% in ECPR patients compared to 18% with conventional CPR. Hospital survival was even higher in our cohort (29%). We recently reported surprisingly good emotional and physical functioning in long-term ECPR survivors (beyond 2 years), which was comparable to the general 55–64 years-old Dutch population^28^. Taken together, we interpret these data as being clearly in favor of ECPR, which we believe not only saves lives, but lives with favorable neurological long-term outcome.

### Limitations

This is a retrospective observational study and therefore contains the risk of selection and reporting bias. Moreover, this is a single-center report and specific processes may influence the presented results.

## Conclusion

Two-thirds of patients with refractory OHCA receiving ECPR had MI Type 1 or 2 warranting immediate revascularization to salvage viable myocardium. Thirty percent of OHCA patients survived to hospital discharge. Considering that ECPR long-term survivors have a favorable neurological outcome, minimizing myocardial scarring and preventing subsequent heart failure appears of paramount importance. We therefore suggest that coronary angiography should be performed in ECPR patients without obvious non-cardiac cause of CA as soon as possible. Ideally, we propose that in-hospital ECPR be performed inside a cardiac catheterization laboratory, followed by coronary angiography. Out-of-hospital ECPR patients should be transferred directly to a catheterization laboratory.
